# Dairy Products Quality from a Consumer Point of View: Study among Polish Adults

**DOI:** 10.3390/nu12051503

**Published:** 2020-05-21

**Authors:** Marta Sajdakowska, Jerzy Gębski, Dominika Guzek, Krystyna Gutkowska, Sylwia Żakowska-Biemans

**Affiliations:** Department of Food Market and Consumer Research, Institute of Human Nutrition Sciences, Warsaw University of Life Sciences (SGGW-WULS), 159C Nowoursynowska Street, 02-787 Warsaw, Poland; jerzy_gebski@sggw.edu.pl (J.G.); dominika_guzek@sggw.edu.pl (D.G.); krystyna_gutkowska@sggw.edu.pl (K.G.); sylwia_zakowska_biemans@sggw.edu.pl (S.Ż.-B.)

**Keywords:** consumer, quality, animal-derived food, yoghurt

## Abstract

The aims of the current study were (a) to deepen the understanding of food quality from animal origin with particular emphasis on dairy products, including yoghurt; (b) to determine the level of acceptance of methods and ingredients used to enhance the quality of food from animal origin; (c) to identify how the perception of animal products quality affects the acceptance of changes in production methods and (d) to identify the projective image of consumers purchasing high-quality yoghurt. The data were collected using a CAPI (Computer Assisted Personal Interview) survey on a sample of 983 consumers. The k-means clustering method (k-means clustering algorithm is an unsupervised algorithm that is used to segment the interest area from the background) was used to identify five clusters of consumers. Moreover, the logistic regression models were used in order to examine the impact of opinions related to the quality of product on acceptance of food production methods. The results showed that food quality is generally perceived by consumers using the following attributes: its freshness, naturalness, production method, as well as appearance, taste and smell, but when it comes to the quality of food from animal origin, convenience, connected with the availability, nutritional value and health benefits is of primary importance. The most accepted production method of high-quality food is animal production that takes into consideration the welfare of farm animals. Results also show that the increase in the level of education among the surveyed people contributed to the acceptance of ensuring welfare of farm animals as a method of increasing food quality while consumers′ openness to new products favored the acceptance of adding health-promoting ingredients to livestock feed. As regards the assessment of the level of acceptance of enhancing food with beneficial ingredients, people for whom health aspects were important declared their willingness to accept such a method of increasing food quality. The research findings can be used to develop educational campaigns as well as marketing communication of enterprises operating on the food market. Furthermore, the results could be used to strengthen the competitive position of food enterprises searching for innovative solutions.

## 1. Introduction

Consumers take various factors into consideration when choosing food; they include taste and freshness as well as naturalness [1,2]. In addition to taste, smell, freshness and naturalness of the product, the following factors also affect consumer choices: the method of food production and processing, ensuring welfare of farm animals and maintaining the health values of food, especially for consumers for whom it is most crucial [3,4]. Moreover, results of studies also indicated that relative advantage, naturalness, novelty and discomfort are the most important factors of the perception of some innovative food products [5]. The literature showed that from a consumer perspective, among other factors, food selection factors are seen as food quality [6,7,8]. This also applies to dairy products [6,7]. The results of studies confirm that consumers take into account a number of attributes associated with quality, so they expect the product to be safe, natural, healthy and generally of high quality [9]. Furthermore, some consumers underlined the role of quality signs, particularly in the field of positioning origin and organic products in the segment of premium prices, emphasizing the authenticity of these products [10].

Moreover, when it comes to functional food, consumer acceptance was also analyzed from the perspective of consumer quality perception of food products. Functional foods provide, from the consumers′ perspective, synergies between healthiness and convenience but may, in the consumers′ opinion, lead to trade-offs between healthiness on the one hand and taste and naturalness on the other hand [11].

The results of studies showed that acceptance of functional dairy products increases among consumers with higher diet/health-related knowledge, as well as with ageing. General interest in health, food-neophobia and perceived self-efficacy seem also to contribute to shaping the acceptance of functional dairy products [12]. Furthermore, products with “natural” matches between carriers and ingredients have the highest level of acceptance among consumers [12,13]. A review by Kaur and Singh indicated that a high level of education and high income greatly influence consumer uptake of functional food, as well as an increased personal health consciousness [14]. Furthermore, results of the research indicate that health benefits and ingredient naturalness are positively valued, but such preferences and valuations depend on an individual′s education, income and food purchase behaviors; thus, naturally occurring nutrients are preferred over fortification [15].

As earlier mentioned, taking into account food safety and food naturalness, the method of food production is also a crucial point. When it comes to the method of food production, including animal welfare-friendly methods, the results of studies among European consumers indicate that public perceptions of farm-animal welfare represent a potentially important driver of consumption behaviors by European consumers [16]. However, some Europeans currently do not think there is sufficient choice of welfare-friendly animal food products in shops and supermarkets [17]. In addition, there is an increasing need to develop policies pertaining to animal production diseases, sustainable intensification and animal welfare, which incorporate consumer priorities as well as technical assessments of farm animal welfare. Consumers may have concerns about intensive production systems and whether animal production disease pose a barrier to consumer acceptance of their increased use [18].

With reference to yoghurt, its nutritional content varies depending on the processing method and the ingredients used. Similar to milk, it is a good source of protein and calcium and may be a source of iodine, potassium and B vitamins [19]. Moreover, some dairy products are fortified with vitamin D [20]. Furthermore, during the past years, interest in yoghurt manufacture has increased for scientific and commercial reasons [21]. Additionally, the functional food market has experienced a tremendous level of growth particularly in yoghurt in the last couple of decades, due to the ease of incorporating pre- and probiotics [22].

Yoghurt still plays an important role in the human diet today due to its pleasant taste and health benefits [22,23]. Moreover, yoghurt is the most-frequently consumed healthy and nutritious food around the world. Therefore, it offers an appropriate potential to provide nutritious ingredients to human diet [24]. Furthermore, the results of research indicate that with respect to the safety and health effects of food products, the probiotic yoghurt is recommended for consumption [25]. Considering the fast evolution of functional yoghurts either at research stage or marketplace, further development would require an accurate measure of quality, safety and efficacy to meet consumers’ expectations on quality and claimable health benefits [26].

Therefore, the aims of the current study were (a) to deepen the understanding of the quality of food from animal origin with particular emphasis on dairy products, including yoghurt; (b) to determine the level of acceptance of methods and ingredients used to enhance the quality of food from animal origin; (c) to identify how the perception of animal products quality affects the acceptance of changes in production methods and (d) to identify the projective image of consumers purchasing high-quality yoghurt.

## 2. Material and Methods

### 2.1. Data Collection Process

The sample in our study (*N* = 983) was drawn from the Social Security addresses database and was representative of the national population in terms of age, gender and the region that consumers lived in. The survey was conducted in each of the 16 voivodships in Poland. After drawing the starting addresses, the random route method was used in the selection of the sample [27,28]. A good number of sampling points were drawn with a probability proportional to population size, for total coverage of the country and for population density. In order to achieve this, the sampling points were drawn systematically from each of the “administrative regional units”, after stratification by individual unit and type of area. They thus represent the whole of Poland as well as the distribution of the resident population. In each of the selected sampling points, a starting address was drawn at random. Further addresses were selected by standard “random route” procedures from the initial address. In each household, a respondent was drawn at random (following the “closest birthday rule”).

The interviews were conducted face-to-face at respondents’ homes by a professional market research agency in accordance with the ESOMAR (European Society for Opinion and Marketing Research) code of conduct using the CAPI (Computer Assisted Personal Interview) technique. All respondents were aged 21+. Only those respondents who met the recruitment criteria, i.e., made their own or cooperative food purchases and declared dairy product consumption, participated in the study.

### 2.2. Description of Questionnaire

The questionnaire used in the study was structured in a few main blocks and covered aspects such as consumer opinion towards: (1) the quality of food, including quality of animal origin food and (2) production methods of animal origin food, formulated into various types of questions:

(A) An open question: *What, in your opinion, shows the quality of food? Please indicate one of the most important attributes;*
(B) Two questions related to the quality of animal origin food (I), including the dairy products (II):
  (I) Below are statements describing food of animal origin. For each statement, how much you agree are indicated on a 1–7 scale, where 1 is the lowest level of compliance and 7 is the highest level of compliance; High-quality animal food is food (1) with the right taste and traditional recipe; (2) preservative free and with a short shelf life, (3) having nutritional value and health benefits, (4) produced in an environmentally friendly area, including taking into account production ensuring welfare of farm animals, (5) of low processing level/derived from an organic production method, (6) which is easy to prepare and easily available in a wide range;
  (II) Please indicate how much you agree with the following statements. Please provide answers on a scale of 1–7, where 1 means “strongly disagree” and 7 means “strongly agree”; (1) I buy dairy products because they have a positive effect on my figure, (2) I buy dairy products because they have a good effect on my children’s health, (3) Quality is important to me when choosing dairy products, (4) I buy dairy products for those members of my family who have health issues;
(C) Questions referring to methods of increasing the quality of food of animal origin are formulated as follows: *To what extent do you accept the following methods of increasing the quality of food of animal origin?* *Please provide answers on a scale of 1–7, where 1 means “definitely do not accept” and 7 “definitely accept”; (1) Adding health-promoting ingredients to livestock feed, (2) Production ensuring welfare of farm animals, (3) Enhancing food products with health-promoting ingredients at the processing stage;*
(D) Questions related to increasing the level of ingredients in dairy products are as follows: *Please specify if you think the content of the ingredients listed below should be increased in dairy products? Where 1 definitely should not be increased, 7 definitely should be increased; (1) Minerals; (2) Fibre, (3) Cholesterol-lowering ingredients, (4) Omega-3 acid, (5) Live bacterial cultures, (6) Protein, (7) Coenzyme Q_10_;*
(E) Questions that allow the determination of projective image of buyers purchasing high-quality yoghurt are formulated as follows: *Who do you think is the most willing to buy high-quality yoghurt? Please give your answer on a scale from 1–7, where: 1 means “Definitely no” and 7 “Definitely yes”, (1) professionally active individuals, (2) sport doers, (3) those looking for nutritional news, (4) the young, (5) the overworked, (6) people with abnormal intestinal motility, (7) cooking lovers, (8) those oriented on the convenience of preparing a meal, (9) bargain hunters, (10) people who are particularly health-conscious.*

### 2.3. Data Analysis

Referring to analysis of the results collected using the open question, the χ^2^ test was applied in order to determine statistically significant differences between the variables (part A of the questionnaire). Moreover, the k-means clustering method was used to identify segments of consumers. In the k-means method (k-means clustering algorithm is an unsupervised algorithm that is used to segment the interest area from the background), in order to increase its efficiency, the average values for individual clusters obtained using the hierarchical method were used as seeds. The statements about the characteristic of food of animal origin were used as segmentation variables (part B I of the questionnaire; Table 1).

Five well-separated clusters were obtained, which was confirmed by both statistics assessing the selection of clusters such as CCC (Cubic Clustering Criteria), pseudo T2 or ANOVA (Analysis of Variance) statistics comparing the average values of variables for individual clusters. Socio-demographic variables such as gender, age, education, subjective assessment of the financial situation and size of the place of residence were used to profile the clusters. The independence χ^2^ test was used to assess the diversity of profile features between clusters.

In all statements analyzed, statistically significant (*p* < 0.05) differences between mean scores particularly clusters have been observed. Additionally, post-hoc test (Waller–Duncan K-ratio *t* Test) was used to compare mean values of opinions between pairs of clusters.

As mentioned, the segmentation analysis made it possible to identify five consumer segments. Clusters have been named according to consumers′ opinions towards statements referring to high quality food of animal origin (Table 1):

(1) “Convenience-oriented” consumers with a high level of compliance with the statement referring to convenience associated with the easy preparation and availability of high-quality food (9.12);
(2) “Uninvolved” consumers with the lowest levels of compliance with most of the statements compared to other segments;
(3) “Health-oriented” consumers with a significantly high level of compliance with the statement describing the acceptance of nutritional value and health values (10.53) compared to other segments;
(4) “Particularly demanding in terms of quality”, consumers with a significantly high level of compliance for most statements referring to high-quality food;
(5) “Neutral but valuing food quality”, consumers declaring relatively high rating levels for most statements but lower rating level for people classified in segment No. 4.

In the second step of data analysis, logistic regression was performed to determine how the perception of animal products quality impacts on:

- The acceptance of adding health-promoting ingredients to livestock feed;
- The acceptance of production ensuring welfare of farm animals;
- The acceptance of enhancing food products with health-promoting ingredients at the processing stage.

Due to the dichotomous nature of dependent variables (accept/not accept), logistic regression models were used [29,30], where dependent variables (regressants) were declarations regarding the acceptance of the above-mentioned 3 methods, and explanatory variables (regressors) were opinions about yoghurts and dairy products expressed in questions, i.e.,: *How much do you agree with the statements describing the quality of dairy products, Who do you think is the most willing to buy high-quality yoghurt? Do you think the content of the ingredients listed below should be increased in dairy products?* The models were built with a stepwise selection of explanatory variables. Only statistically significant variables at the significance level α = 0.05 were included in the models. The statistical analysis was carried out using IBM SPSS Statistics, version 25.0 (IBM Corp., Armonk, NY, USA) and SAS 9.4 statistical package (SAS Institute, Cary, NC, USA).

## 3. Results

### 3.1. Profile of the Total Sample and Perception of Food Quality 

The detailed socio-demographic characteristic of the sample and segments identified is included in Table 2.

Results of the study show that the quality of food of animal origin is connected with the ease of preparation, availability, as well as nutritional value and health benefits (Table 1), but when it comes to the food quality in general, it is perceived by consumers mainly through the following attributes: its freshness, naturalness, production method, as well as appearance, taste and smell (Table 3).

Results also show (Table 2 and Table 3) that in segment No. 1 (“Convenience-oriented”; *N* = 208; 21%) the largest share of opinions indicate that the quality of food of animal origin is evidenced by its naturalness, production method, but also its freshness. This segment was characterized by the largest share of middle-aged people (45–54 years).

In segment No. 2 (“Uninvolved”; *N* = 172; 18%), the largest share of the food quality is reflected in its freshness, naturalness and the production method. This segment had a relatively high share of people with low levels of education.

In segment No. 3 (“Health-oriented”; *N* = 218; 22%), the highest share of answers indicating that the quality of food is reflected in its freshness was recorded. There was also a relatively large share of opinions indicating that the quality of food of animal origin is reflected in its naturalness and production method. The additional attribute of food quality that was mentioned by people was lack of preservatives in the product.

In segment No. 4 (“Particularly demanding in terms of quality”; *N* = 159; 16%), there was a relatively large share of opinions indicating that the quality of food of animal origin is reflected in its composition and nutritional values as well as appearance, taste and smell. The segment had the largest share of indications that the determinant of food quality is its price. This segment had the relatively high share of people with low levels of education.

In segment No. 5 (“Neutral but valuing food quality”; *N* = 226; 23%), among the indications characterizing the quality of food of animal origin, mainly its freshness, naturalness, method of production as well as the aspects referring to composition and nutritional value of food were mentioned. 

### 3.2. Methods of Improving Quality of Animal Origin among The Clusters of Consumers

The results show that among the methods of increasing the quality of food of animal origin, consumers scored the highest for the production method ensuring welfare of farm animals compared to the other two methods. Comparison of the segments show that respondents from segment No. 3 (“Health-oriented”) displayed significantly lower acceptance, compared to other segments regarding the method of adding health-promoting ingredients to livestock feed (Table 4). 

In addition, scores on production ensuring welfare of farm animals by respondents in segment No. 5 (“Neutral but valuing food quality”) was significantly lower compared to consumers in segments 1, 2 and 3, while respondents from segment No. 4 significantly displayed lower acceptance of this type of production compared to other segments. Scores on enhancing food products with health-promoting ingredients at the processing stage by respondents from segment No. 4 (“Particularly demanding in terms of quality”) was significantly higher compared to other segments, and respondents from segment No. 3 (“Health-oriented”) had significantly lower acceptance of this type of enrichment compared to other segments (Table 4).

Regarding the increase of some ingredients in dairy products, live bacterial cultures and cholesterol lowering ingredients were the most important types of ingredients that should be increased in the consumers′ opinion in dairy products (Table 5). Consumers in segment 2 (“Uninvolved”), which was also significantly higher compared to segments 3, 4 and 5, agreed that the content of cholesterol-lowering ingredients should be increased in dairy products. “Uninvolved” also declared a significantly higher level of acceptance compared to other segments, in terms of increasing the content of live bacterial cultures and coenzyme Q_10_ in dairy products. In the case of increasing the content of minerals and increasing the fiber content in dairy products, the “Uninvolved” significantly agreed with this opinion in comparison to “Health-oriented” and “Particularly Demanding in Terms of Quality”. On the other hand, “Health-oriented” segment displayed the lowest degree of acceptance with regards to increasing the level of Omega-3 acid, proteins and Coenzyme Q_10_ in dairy products compared to other segments (Table 5).

The next part of the study was aimed at determining the image of consumers of high-quality yoghurt (Table 6). Respondents perceived consumers of high quality yoghurts referring to two main aspects: (1) health and (2) physical activity.

Respondents from segment No. 4 (“Particularly demanding in terms of quality”) in the least degree compared to the other segments agreed with the opinion that such consumers are people who can be characterized as: doing sports, young people, as well as people with abnormal intestinal motility and people who are particularly health-conscious.

### 3.3. Impact of Selected Attributes on Methods of Improving Quality of Animal Origin Food

In the next stage of the study, the extent in which consumers would accept 3 methods aimed at increasing the level of food quality was assessed.

The rise in the acceptance of opinion that the content of live bacterial cultures in dairy products should be increased resulted in a 47% increase in the willingness of accepting production ensuring welfare of farm animals (OR: 1.47; 95% CI: 1.14–1.90), while maintaining other model parameters at a constant level. The level of education had an impact on the acceptance of production, ensuring the welfare of farm animals. The higher the level of education, the greater the willingness of this acceptance. This willingness in the case of people with secondary education increased four times compared to people with primary education (OR: 4.06; 95% CI: 1.82–12.93). In the case of higher education, the willingness of acceptance increased more than 10-fold (OR: 10.25; 95% CI: 1.95–22.49) in relation to people with primary education (Table 7).

The rise (by 1 point) in acceptance of the opinion that the content of minerals in dairy products should be increased resulted in a 21% increase in the willingness of acceptance of adding health-promoting ingredients to livestock feed (OR: 1.21; 95% CI: 1.06–1.37). The rise in importance of the opinion that high-quality yoghurts are bought by people involved in sports resulted in a 27% decrease in willingness of acceptance of adding health-promoting ingredients to livestock feed (OR: 0.73; 95% CI: 0.58–0.91). The increase in the rank that high-quality yoghurts are bought by those looking for novelty foods increased by 19% compared to the willingness of acceptance of adding health-promoting ingredients to livestock feed (OR: 1.19; 95% CI: 1.02–1.40). People declaring that quality is important to them when choosing dairy products showed a 19% lower willingness of accepting the addition of health-promoting ingredients to livestock feed (OR: 0.81; 95% CI: 0.65–0.99) along with increasing the rank of this opinion by 1 level (Table 8).

The rise in importance of the opinion that the content of cholesterol-lowering ingredients should be increased in dairy products resulted in a 29% increase in the willingness to accept enhancing food products with pro-health ingredients at the processing stage (OR: 1.29; 95% CI: 1.13–1.47). Obviously, while maintaining the remaining model parameters at a constant level. The increase in the rank referring to opinion that high-quality yoghurts are bought by the professionally active individuals resulted in a 30% decrease in the level of acceptance of enhancing food products with pro-health ingredients at the processing stage (OR: 0.70; 95% CI: 0.56–0.86). The increase in the rank of the opinion that high-quality yoghurts are bought by people with abnormal intestinal motility gave a 31% greater willingness of accepting enhancing food products with health-promoting ingredients at the processing stage (OR: 1.31; 95% CI: 1.06–1.60). Similar results were seen in the responses that high-quality yoghurt is bought by those looking for price bargains. In this case, the willingness of accepting enhancing food products with health-promoting ingredients at the processing stage increased by 24% (OR: 1.24; 95% CI: 1.08–1.41). The willingness to accept enhancing food products with health-promoting ingredients at the processing stage decreased by 26% in the case of persons agreeing with the opinion that quality is important for them when choosing dairy products (OR: 0.74; 95% CI: 0.60–0.90). The rise in the rank of the opinion that *I buy high-quality dairy products for those family members who have health issues* resulted in a 17% increase in enhancing food products with health-promoting ingredients at the processing stage (OR: 1.17; 95% CI: 1.04–1.33) (Table 9).

## 4. Discussion

The study presents the results of a survey on a representative sample of consumers. The analysis of the obtained results indicated that the quality of animal origin food with particular emphasis on dairy products is of great importance to consumers, and they are willing to accept new methods of production and ingredients in dairy products.

### 4.1. The Food Quality from a Consumer Point of View

Our study revealed that the consumer’s perception of food quality differ among segments. Furthermore, the high-quality products of animal origin were perceived by consumers in various ways depending on the segment, so the results are consistent with previous studies stating that understanding the personal and context specific influences on consumer quality perceptions is important in developing products that meet consumer needs [31].

In general, referring to the aspect of food quality, the results showed that taking into account the consumer segments, consumers in segment No. 4 (“Particularly demanding in terms of quality”) slightly agreed with the opinion that the group of people buying yoghurts perceived as high-quality yoghurts includes people involved in sports, young people, people with abnormal intestinal motility and those concerned about their health. Analysis of the research findings showed that consumers in segment 2 (“Uninvolved”) showed high levels of indications in terms of increasing the level of health-promoting ingredients, which may suggest that despite a relatively indifferent position on food quality compared to other consumer segments, these people were interested in increasing the amount of selected ingredients, and at the same time, it may prove that consumers expect producers and processors to take appropriate action on their behalf to improve their health. This is reflected in the studies by other authors, which emphasized the importance of health as a value influencing the acceptance of specific type of food [32]. Moreover, referring to milk products, the totality of available scientific evidence supports the fact that intake of milk and dairy products contributes to meet the nutrient recommendations and may protect against the most prevalent chronic diseases, whereas, very few adverse effects have been reported [33]. Furthermore, lactose malabsorption is widespread in most parts of the world, with wide variation between different regions and an overall frequency of around two-thirds of the world’s population [34].

### 4.2. The Acceptance of Production Ensuring Welfare of Farm Animals

Our study assessed the level of acceptance of methods used to increase the food quality and selected factors that may affect the level of this acceptance among consumers. In general, animal welfare is the credence quality attribute [7] that is of great interest to consumer. The results showed that the increase in education contributed to the acceptance of production ensuring welfare of farm animals as a way of increasing the level of healthy ingredients in food. The results of other studies indicate that individuals involved in health and/or sustainable eating are more likely to be better educated than those who are not involved [35]. This may be due to a greater awareness of ensuring adequate welfare of farm animals (and/or probably due to the sensitivity of this group of people to animal suffering) [36]. Results also show that the use of appropriate production ensuring welfare of farm animals as a method of increasing food quality is also accepted by consumers who willingly accept increasing the content of live bacterial cultures in dairy products. This can be associated with the positive consumer perception of yoghurt through the aspect referring to health issue, which is confirmed by the studies of other authors [26,37].

The results of other studies indicate that consumers with a higher income and higher education were willing to pay more for farm animal welfare [38]. The results of studies revealed also that referring to animal welfare, the provision of additional information significantly increased the intention to purchase higher than the conventional welfare products. The empathy measures revealed that younger participants, females and those with lower household incomes all had significantly higher AES (Animal Empathy Score). Moreover, this score was associated with the intent to purchase higher welfare products [39]. However, some studies showed that consumers are, in general, unaware about welfare issues at the farming level [40,41]. In addition, an analysis of the results of surveys performed under Euro barometer 2019 [42] indicated that the most important factors for Europeans when buying food are where the food comes from (53%), cost (51%), food safety (50%) and taste (49%). Nutrient content is considered slightly less important (44%), while ethics and beliefs (e.g., considerations of animal welfare, environmental concerns) rank lowest in importance (19%) [42]. However, Vanhonacker and Verbeke [43] noticed that the role of information on animal welfare as well as the type of consumer to whom this information is presented is important when making purchasing decisions. Moreover, in their opinion, the issue involves acknowledging that not everyone has the same level of interest in animal welfare or in purchasing higher welfare products. Furthermore, not all individuals with an interest in higher welfare products share the same motivation. Information sharing should thus be adjusted to specific target segments [43]. On the other hand, the results of more recent research [39] suggest that concern for the welfare of animals farmed for food remains high and continues to grow. Moreover, this research indicates that providing consumers with descriptive signals referring to the welfare condition at the point-of-purchase can boost welfare purchase intentions [39].

### 4.3. The Acceptance of Adding Health-Promoting Ingredients to Livestock Feed

The second method of increasing the food quality that was accessed in the survey, was adding health-promoting ingredients to livestock feed. It was observed that consumer acceptance of opinions on increasing mineral components at the same time inclines them to accept adding health-promoting ingredients to livestock feed. The results of other studies showed that in the area of animal nutrition, the opportunity for improving quality is by adding health-promoting ingredients to livestock feed containing additives such as vitamins, vitamin-like compounds, minerals including trace elements, fatty acids, probiotics and other bioactive compounds [44,45].

The results also showed that with the increase in acceptance of novelty on the food market, the level of acceptance of the production method which entails adding health-promoting ingredients to livestock feed increases. This may be associated with generally greater openness to changes in the food market and a higher level of acceptance of changes in this market in relation to some consumer groups [46]. When it comes to Polish consumers, in general, the new generation of Poles is relatively more open to new food products due to the wide range of food products available on the free market. Furthermore, the group of well-educated consumers with a higher level of income has increased in size, and this includes people interested in knowledge of a product’s nutritional value and its impact on health [47]. Results showed that people for whom quality was important when choosing dairy products and people doing sports did not accept this method of improving quality, which confirms their special interest in health aspects and possible consequences and/or concerns related to the consumption of this category of food.

### 4.4. The Acceptance of Enhancing Food Products with Health-Promoting Ingredients at The Processing Stage

Among various food choice motives, health is thought to be the highly important factor in consumer opinion [3]. Our results showed that with regard to the third method of increasing the food quality, people seeking the possibility of lowering blood cholesterol levels, people who believe that “high-quality yoghurt is bought by people with abnormal intestinal motility” and those who bought yoghurt for members of their families with health issues expressed their willingness to accept increasing the level of ingredients at the food processing stage.

The results of other studies indicated that respondents with a history of familial diseases were more likely than others to have consumed margarine with plant sterol, fruit juices fortified with vitamin C, and breakfast cereals fortified with vitamins and minerals [48]. It was also found in other studies that consumers who considered health, sensory appeal, natural content, and ethicality to be important factors in their food choices and were concerned about their health, considered yoghurts which were reasonably sour, thick, and genuine in flavor to be more pleasant [3]. Results of similar studies among consumers regarding functional food showed that food benefits were more positively evaluated when attached to a more attractive carrier (e.g., yoghurt). Moreover, benefits of improving the body’s natural defense system were most favored by all groups of surveyed consumers while benefits about specific diseases were suitable to tailor for certain groups [13]. Generally, our results are consistent with other studies showing that claims referring to prevention of the diseases are accepted by the consumers [49,50].

It should be emphasized, however, that the results of our study showed that consumers paying special attention to quality were afraid of the above-mentioned methods, declaring their low level of acceptance, which may indicate that they believe there are some concerns related to increasing the level of some ingredients or the fear of using selected methods are not well known to them, which may be associated with so-called food technology neophobia [51]. Research shows that the majority of consumers have relatively little knowledge about the technologies used in food production [52]. However, when it comes to advertising and marketing to consumers about new technologies, campaigns that incorporate convenience, naturalness, taste and benefit for the consumer could have a positive impact on consumer food choices, particularly when the message is concise and from trusted sources [53].

## 5. Conclusions

The significant role of food quality in decisions taken on the food market, as well as the availability of products with special health benefits, encourages the assessment of consumer behavior in relation to food perceived as the high-quality food and learning about consumer opinions on products that have a positive impact on health.

The results show that in general, food quality is perceived by consumers mainly through the following attributes: freshness, naturalness, production method, as well as appearance, taste and smell, while when it comes to the quality of food of animal origin, convenience connected with availability, nutritional value and health benefits are of primary importance.

The most accepted production method of high-quality food is animal production with respect to the welfare of farm animals. It is a particularly important aspect, because animal welfare is an important element of sustainable development, including food consumption and human diet and can positively contribute to food quality. Results also show that the increase in the level of education of the surveyed people contributed to the acceptance of production, ensuring welfare of farm animals as a method of increasing food quality.

With regard to the acceptance of other methods aimed at increasing the content of health-promoting ingredients, it should be emphasized that consumer openness to new products favored the acceptance of adding health-promoting ingredients to livestock feed. Regarding the assessment of the level of acceptance by consumers of enhancing food with beneficial ingredients at the processing stage, people for whom health aspects were important declared their willingness to accept such a method of increasing food quality.

Moreover, the research findings can be used to develop educational campaigns as well as in marketing communication of enterprises operating on the food market. When it comes to the educational campaigns, there are opportunities to increase the level of consumer awareness referring to animal welfare that is still not considered an issue for many of the Central and Eastern European citizens. Moreover, to strengthen the competitive position of food enterprises, an important point could be the development of the food products labelled with information on animal production, ensuring welfare of farm animals.

In addition, in terms of information on the methods of improving quality communicated to consumers, for some people, the manner in which this information is presented will play an important role, as well as the level of awareness of the recipients to whom it was addressed, and the possible health consequences they perceive. Therefore, the observed impact of the level of education as well as the health benefits of accepting some methods of increasing food quality should be used on the food market. This aspect could be particularly important according to the development of health and nutrition claims.

The present study fills the relevant research gaps regarding enhancing the quality of food from animal origin and explores methods referring to increasing the level of food quality, providing a new perspective to food industry and the scholars. On the one hand, there are some differences regarding the consumer acceptance referring to methods of enhancing food quality. On the other hand, the determinants that impact the level of acceptance are also various. Future research studies should concentrate on investigating and developing the level of consumers’ acceptance of new production methods. Nevertheless, it should be noted that our results indicated the main directions regarding the acceptance of the used productions methods as well as designated the possible changes that may be accepted by consumers. 

## Figures and Tables

**Table 1:** Statements used as segmentation variables regarding the characteristics of high-quality products of animal origin.

Attributes	Mean	Convenience-Oriented 1	Uninvolved 2	Health-Oriented 3	Particularly Demanding in Terms of Quality 4	Neutral but Valuing Food Quality 5	*p*-Value
Easy preparation and availability	6.79	9.12 a	2.20 d	6.79 c	6.50 c	8.30 b	<0.0001
Nutritional value and health benefits	5.95	2.82 d	2.37 e	10.53 a	7.84 b	5.76 c	<0.0001
Processing, organic production	4.51	2.73 d	2.99 d	3.66 c	7.18 a	6.23 b	<0.0001
Tradition and taste	4.17	2.64 c	2.71 c	2.71 c	8.84 a	4.81 b	<0.0001
Lack of preservatives and shelf life	4.12	2.72 c	3.07 c	3.01 c	8.10 a	4.47 b	<0.0001
Environment and animal rights	3.92	2.60 d	2.25 d	2.98 c	7.10 a	5.06 b	<0.0001

One-Way ANOVA (Analysis of Variance), *p* < 0.05; a, b, c, d, e—Means with the same letter are not significantly different in Waller-Duncan test.

**Table 2:** Socio-demographic characteristics of the consumers surveyed (*N* = 983, Poland) (%).

Variables	Total Sample (%)	Convenience-Oriented *N* = 208; 21% 1	Uninvolved *N* = 172; 18% 2	Health-Oriented *N* = 218; 22% 3	Particularly Demanding in Terms of Quality *N* = 159; 16% 4	Neutral But Valuing Food Quality *N* = 226; 23% 5	*p*-Value
Gender							0.7462 *
Female	51.41	54.85	52.07	51.15	51.01	48.15	
Male	48.59	45.15	47.93	48.85	48.99	51.85	
Age							0.5216 *
21–27	16.30	15.05	15.98	13.36	19.46	18.52	
28–34	15.99	16.02	14.20	12.90	20.13	17.59	
35–44	18.18	17.48	15.98	17.97	19.46	19.91	
45–54	20.06	24.76	21.30	20.74	14.77	17.59	
55–64	18.81	18.45	21.30	21.66	17.45	15.28	
65–75	10.66	8.25	11.24	13.36	8.72	11.11	
Education							0.0102
Primary, lower secondary, vocational	47.75	41.26	55.03	43.78	54.36	47.69	
Secondary	37.10	38.35	36.09	39.17	36.91	34.72	
Higher	15.15	20.39	8.88	17.05	8.73	17.59	

* Differences between groups not significant (χ^2^ test, *p*-value > 0.05).

**Table 3:** Attributes describing food quality in consumer reviews (%).

Attributes	Number of Indications	%	Convenience-Oriented *N* = 208; 21% 1	Uninvolved *N* = 172; 18% 2	Health-Oriented *N* = 218; 22% 3	Particularly Demanding in Terms of Quality *N* = 159; 16% 4	Neutral But Valuing Food Quality *N* = 226; 23% 5	*p*-Value
Freshness	197	20.04	19.71	19.19	27.98	7.55	22.12	<0.0001
Naturalness, production method	162	16.48	20.19	15.70	18.35	7.55	18.14	
Appearance, taste, smell	121	12.31	17.31	10.47	8.26	13.84	11.95	
Composition, nutritional values	113	11.50	10.10	12.79	7.34	15.09	13.27	
Preservative-free	100	10.17	7.69	10.47	12.84	8.81	10.62	
Price	62	6.31	3.85	4.65	1.38	13.84	9.29	
Producer	47	4.78	4.81	6.98	5.5	4.40	2.65	
Shelf life	42	4.27	2.88	4.65	5.96	5.66	2.65	
Quality mark	38	3.87	3.85	4.65	5.05	6.29	0.44	
Origin	37	3.76	6.25	2.91	3.67	3.77	2.21	
No answer/I do not know	64	6.51	3.37	7.56	3.67	13.21	6.64	

Test of independence χ^2^ *p* < 0.05.

**Table 4:** The level of acceptance of methods to increase food quality of animal origin in the opinion of respondents.

Selected Methods of Increasing Food Quality	Mean	Convenience-Oriented*N* = 208; 21%1	Uninvolved *N* = 172; 18% 2	Health-Oriented *N* = 218; 22% 3	Particularly Demanding in Terms of Quality *N* = 159; 16% 4	Neutral But Valuing Food Quality *N* = 226; 23% 5	*p*-Value
Animal production ensuring welfare of farm animals	5.90	6.21 a	6.32 a	6.18 a	5.13 c	5.60 b	<0.0001
Adding health-promoting ingredients to livestock feed	4.14	4.32 a	4.35 a	3.39 b	4.43 a	4.35 a	<0.0001
Enhancing food products with health-promoting ingredients at the processing stage	3.85	3.89 b	4.21 b	2.84 c	4.61 a	4.00 b	<0.0001

One-Way ANOVA, *p* < 0.05; a, b, c—Means with the same letter are not significantly different in Waller–Duncan test.

**Table 5:** Consumers′ opinion on increasing in dairy products the level of ingredients that have a positive impact on health.

Food Ingredients Whose Level Should Be Increased	Mean	Convenience-Oriented *N* = 208; 21% 1	Uninvolved *N* = 172; 18% 2	Health-Oriented *N* = 218; 22% 3	Particularly Demanding in Terms of Quality *N* = 159; 16% 4	Neutral But Valuing Food Quality *N* = 226; 23% 5	*p*-Value
Live bacterial cultures	4.88	5.03 b	5.51 a	4.51 c	4.78 bc	4.76 bc	<0.0001
Cholesterol lowering ingredients	4.84	4.97 ab	5.27 a	4.55 b	4.67 b	4.81 b	0.0101
Minerals	4.76	4.85 ab	5.20 a	4.37 c	4.70 bc	4.78 ab	0.0024
Fiber	4.69	4.85 ab	5.07 a	4.41 c	4.49 bc	4.69 abc	0.0142
Omega-3 acid	4.50	5.67 a	5.00 ab	4.01 c	4.48 b	4.51 b	0.0006
Protein	4.42	4.35 b	4.93 a	3.89 c	4.75 ab	4.40 b	<0.0001
Coenzyme Q_10_	4.32	4.41 b	5.03 a	3.73 c	4.31 b	4.35 b	<0.0001

One-Way ANOVA, *p* < 0.05; a, b, c—Means with the same letter are not significantly different in Waller–Duncan test.

**Table 6:** Projective image of high-quality yoghurt consumers.

High-Quality Yoghurts Are Purchased by	Mean	Convenience-Oriented *N* = 208; 21% 1	Uninvolved *N* = 172; 18% 2	Health-Oriented *N* = 218; 22% 3	Particularly Demanding in Terms of Quality *N* = 159; 16% 4	Neutral But Valuing Food Quality *N* = 226; 23% 5	*p*-Value
those who are particularly health-conscious	5.95	6.25 a	6.30 a	5.94 b	5.21 c	5.89 b	<0.0001
those with abnormal intestinal motility	5.82	6.03 a	6.07 a	6.04 a	5.15 c	5.71 b	<0.0001
sport doers	5.76	6.03 a	5.89 a	5.94 a	5.18 c	5.62 b	<0.0001
the young	5.55	5.57 b	5.86 a	5.69 ab	5.08 c	5.45 b	<0.0001
professionally active	5.53	5.68 a	5.71 a	5.10 b	5.67 a	5.44 a	0.0005
those looking for nutritional novelties	5.36	5.48 ab	5.73 a	5.17 bc	5.09 c	5.34 bc	0.0019
those oriented on the convenience of preparing a meal	5.33	5.55 a	5.54 a	5.30 a	4.94 b	5.24 ab	0.0030
the overworked	5.23	5.34 a	5.28 ab	5.33 a	4.90 b	5.20 ab	0.1103
cooking lovers	4.66	4.50 bc	4.97 a	4.33 c	4.80 ab	4.76 abc	0.0156
bargain hunters	4.53	4.11 c	4.60 ab	4.37 bc	4.81 a	4.78 ab	0.0030

One-Way ANOVA, *p* < 0.05; a, b, c—Means with the same letter are not significantly different in Waller–Duncan test.

**Table 7:** Prediction of the acceptance of production ensuring welfare of farm animals.

Variable	e^β^	β	95% Wald CI		*p*-Value
Intercept		0.142			0.8728
Independent variables (regressors):					
Increasing the content of live bacterial cultures in dairy products	1.47	0.390	1.14	1.90	0.0024
Basic vocational education vs. primary education	2.32	0.839	0.56	9.51	0.2441
Secondary education vs. primary education	4.06	1.400	1.82	12.93	0.0447
Higher education vs. primary education	10.25	2.327	1.95	22.49	0.0250

e^β^ (OR)—point estimate; β—estimate; 95% Wald CI—95% Wald confidence interval.

**Table 8:** Prediction of acceptance of adding health-promoting ingredients to livestock feed.

Variable	e^β^	β	95% Wald CI		*p*-Value
Intercept		2.248			0.006
Independent variables (regressors):					
Increasing the mineral content in dairy products	1.21	0.187	1.06	1.37	0.004
Purchase of high-quality yoghurt by people involved in sport	0.73	−0.315	0.58	0.91	0.004
Purchase of high quality yoghurts by people seeking nutrition novelties	1.19	0.177	1.02	1.40	0.031
Quality is important when choosing dairy products	0.81	−0.212	0.65	0.99	0.044

e^β^ (OR)—point estimate; β—estimate; 95% Wald CI—95% Wald confidence interval.

**Table 9:** Prediction of acceptance of enhancing food products with health-promoting ingredients at the processing stage.

Variable	e^β^	β	95% Wald CI		*p*-Value
Intercept		0.115			0.8825
Independent variables (regressors):					
Increasing the content of cholesterol-lowering ingredients in dairy products	1.29	0.258	1.13	1.47	0.0001
Purchase of high-quality yoghurt by professionally active people	0.70	−0.355	0.56	0.86	0.0010
Purchase of high-quality yoghurt by people with abnormal intestinal motility	1.31	0.267	1.06	1.60	0.0104
Purchase of high quality yoghurts by people looking for bargains	1.24	0.212	1.08	1.41	0.0021
Quality is important when choosing dairy products	0.74	−0.304	0.60	0.90	0.0032
Purchase of high-quality dairy products for family members who have health issues	1.17	0.162	1.04	1.33	0.0101

e^β^ (OR)—point estimate; β—estimate; 95% Wald CI—95% Wald confidence interval.
